# Association between Awareness of Patient Rights and Patient’s Education, Seeing Bill, and Age: A Cross-Sectional Study

**DOI:** 10.5539/gjhs.v6n3p55

**Published:** 2014-02-17

**Authors:** Mahbobeh Yaghobian, Sima Kaheni, Mahmonir Danesh, Farideh Rezayi Abhari

**Affiliations:** 1 Nursing and Midwifery Management Department, Mazandaran University of Medical Sciences, Sari, Iran; 2 Pediatric Nursing Department, Mazandaran University of Medical Sciences, Sari, Iran; 3 Midwifery Department, Mazandaran University of Medical Sciences, Sari, Iran

**Keywords:** awareness, bill of patient rights, teaching hospital, Mazandaran, Sari, Iran

## Abstract

**Background:**

Considering the controversial results of previous reports on awareness of bill of patients’ rights in different regions, as well as the fact that no report is available on the awareness of patients of their rights in teaching hospitals of Sari, we conducted the present study.

**Materials and Methods:**

This is a cross-sectional Study conducted in teaching hospitals of Sari in 2011. The study population consisted of 336 patients recruited from 4 hospitals affiliated with Mazandaran University of Medical Sciences in Sari, through calculating the selection quota of each hospital. Data were collected through face-to-face interview on discharge, using a two-section questionnaire based on the bill of patient rights and with verified reliability and validity. Data analysis was accomplished on SPSS soft- ware version 10.

**Results:**

55.4% of patients were women and 44.6% were men. The patients’ mean age was 40.93 ± 15.04 years and the mean length of stay was 4.6 ± 3.34 days. Most patients had elementary education (36%) or were illiterate (25%). The majority (63.4%) had not seen the bill of patients’ rights. 58.9% had poor knowledge, 12% had intermediate knowledge, and 29.1% had good knowledge. As for the articles of the bill, the poorest awareness correlated to the 9^th^ article (the right to participate or refrain from participating in research). We found a significant relationship between awareness of the bill, and the patient’s education, seeing the bill, and age (p<0.0005).

**Conclusion:**

The results of the present study indicate that patients are not sufficiently aware of their rights, and this problem requires comprehensive planning to be resolved.

## 1. Background

Nowadays, healthcare systems in many countries have defined certain rights for patients, and the healthcare providers are obligated to abide by these rights when providing service (Joolaee, 2008). The notion of patient rights has been developed on the basis of concept of the person, and the fundamental dignity and equality of all human beings recognized in the Universal Declaration of Human Rights in 1948. Since, numerous declarations and professional ethical codes have sought to ensure the protection of fundamental human rights and to promote the humanitarian treatment of all patients (Välimäki, 2009).

Despite the efforts to prepare and spread the charter of patient rights, studies from different countries have reported infringement of patients rights, such as not aware of the regulation about patient rights (Ducinskiene, 2006; Zülfikar, 2001), ethical and legal implication (Rowe, 2013) observance of medical ethics is inadequate in hospitals (Humayun, 2008) most psychiatric hospitals in Shanghai have no Medical Ethics Committee (Su, 2012).

In Turkey, only 9% of patients were reported to be aware of their rights (Kuzu, 2006). In China, only 9.5% of medical staff thought that patient treatment would be compromised by refusal to participate (Liang, 2012).

In Poland, patients had the least awareness about the form of giving consent (42.9%) or refusal consent.

To treatment (50.5%), and the doctors’ right to refuse the presence of a person close to the patient during treatment (16.4%) while over 80% respondents were aware of their rights such as: choose a treating physician, refusal of the proposed treatment, the choice of the place (Krzych, 2013).

Patient rights constitute an essential component for determining standards of clinical governance. The bill of patient rights was first devised in 2002 in Iran and mandated by the Deputy of Health at Ministry of Health and Medical Education. According to this mandate, all healthcare centers were required to pitch the contents of the bill in a conspicuous spot. In October 2009, the final text of the charter of patient rights was prepared in 5 main axes and 37 articles alongside, insight, value and a final article, and was dispatched to all subsidiaries of the Ministry of Health. The 5 axes consisted of the right to receive appropriate service, the right to receive appropriate and sufficient information, the right of autonomy and free will, and right of privacy and confidentiality, and the right of access to a competent system of complaints, each axis containing 14, 4, 7, 9, and 3 articles, respectively (Parsapour, 2011)

Despite the efforts by the Deputy of Health at Ministry of Health and Medical Education, researcher had not seen bill of patients’ rights until two mounts November and December 2011 in 5 main axes and 37 articles in 4 hospitals affiliated with Mazandaran University of Medical Sciences.

Unfourtunatly, I had seen bill of patients’ rights in 10 articles (first devised in 2002) were installed in the hallway 4 hospitals.

As patients’ awareness varies from one region to another, as well as the facts that no study has been conducted so far to address patients’ knowledge of their rights in teaching hospitals of Sari, and the patients’ rights are poorly observed in hospitals associated with Mazandaran University of Medical Sciences (only 14.59%) (Kazemnezhad, 2013), and patients’ awareness will facilitate the observance of patients’ rights (Joolaee 2006), and this is among the research priorities of the Deputy of Treatment, we conducted the present study to measure the rate of patients’ awareness of their rights in teaching hospitals of Sari.

### 1.1 Objectives

The study aims to investigate: (1) to measure the rate of patients awareness of their rights in teaching hospitals of Sari; (2) correlation between background information and patients awareness of their rights.

## 2. Materials and Methods

### 2.1 Study Design

This study used a cross-sectional design. Data was collected using structured interview face-to-face.

### 2.2 Sample and Setting

The sample consisted of 336 patients from 4 teaching hospitals affiliated with Mazandaran University of Medical Sciences. Using the equation (d=0.05, P=0.32 (Mossadeghzadeh, 2006), Z=1.96) the sample size was calculated 336 individuals.

The distribution of samples in each hospital was determined through calculating the selection quota for each hospital (dividing the number of admission per month in the hospital by the overall admissions in the population study): Imam Khomeini hospital was assigned 215 individuals, Fatemeh Zahra hospital was assigned 70 individuals, Zare hospital was assigned 27 individuals, and Bu Ali hospital was assigned 24 individuals.

### 2.3 Procedure

The study was approved by the research center for Traditional and Alternative Medicine and Study Center for Medical Ethics and History Mazandaran University of Medical Sciences (NO: 90-142), to the mentioned hospitals in two months (November and December 2011) with the introduction letter from the Deputy of Research, and interviewed the discharging patients with permission of the hospital managers.

Stage simple random sampling:

- Researcher referred to the hospital discharged, with letters of introduction from Mazandaran University of Medical Sciences
- Numbered list of patients being prepared for discharged
- Maximum daily interviews were conducted with 10 patients
- Patients were selected by lottery method
- All of wards 4 hospitals except: the emergency department, oncology, pediatrics and psychiatric wards

If the patient was unable to respond, the patient’s next of kin would be interviewed. All patients expressed their consent prior to answering the questions. They were reassured that their anonymity will be preserved.

### 2.4 Measures

Data was collected using a questionnaire with two parts: the first part dealt with demographic information (sex, age, insurance type, length of stay, education, and two questions about the bill of patient rights: see the bill of patients’ rights, and source of information on the contents of the bill), and the second part contained 10 questions addressing the content of the bill (Right of anonymous treatment, right to know the name of physicians, nurses etc, right to know about the treatment process, right to know about complications of therapy, right of discharge with free will, right of privacy, right of confidentiality by physicians and permission, right of access to physicians etc, right to participate in or refrain from a research, right to learn about insurance coverage.

Questionnaires were completed on discharge through face-to-face interviews. Although the questions were prepared using the text of the bill, we confirmed the content validity of the questionnaire with opinions from the committee of research ethics at Mazandaran University of Medical Sciences. For reliability, in pilot study with 20 patients being discharged were interviewed. Cranach’s alpha questions (0/76) were obtained.

The second part of the questionnaire was designed with 10 close-ended questions with yes and no options, the former scored 1 and the latter scored 0. The minimum score was 0 and the maximum was 10. Scores ranging between 0-3 were deemed Poor, 4-6 deemed Intermediate, and more than 7 were deemed good.

### 2.5 Analysis

Collected data were analyzed using descriptive and inferential statistics on SPSS (statistical pakage for the social science, version 10, Inc., Chicago, IL, USA).

Descriptive statistics as mean scores, standard deviations and frequencies were used to describe the demographic characteristics of the patients along with study variables.

Two inferential statistics were used including pearson correlation coefficient (pearson r) to test the correlation between selected factors and scores patients awareness of their rights and dependent T-Test to detect relationship between age, length of stay, and their awareness scores.

## 3. Results

The results indicate that out of 336 individuals, 55.4% were women and 44.6% were men. Most patients were educated below high school level (36%); 25% were illiterate and only 4.2% had a bachelor’s degree. Years of education varied from 0 to 16 years (mean: 6.82 ± 5.29). The mean age of participants was 40.93 ± 15.04 years. The participants were recruited from 4 teaching hospitals of Sari (Mazandaran University of Medical Sciences) based on the number of admissions; thus, the majority of cases (63.7%) pertained to Imam Khomeini Hospital in internal medicine, general surgery, gynecologic surgery, and obstetric wards, 21.1% belonged to the cardiology and post CCU wards of Fatemeh Zahra hospital, 7.1% were selected from the internal medicine and surgery wards of Bu Ali hospital, and 0.8% were recruited from the burns and reconstructive medicine ward of Zare hospital.

As for insurance type, the majority (34.2%) had Medical Services insurance, followed by Social Security insurance (32.7%), Welfare Committee insurance (16.7%), Rural insurance (13.7%) and others (2.7%). The mean length of hospital stay was 4.6 ± 3.34 days.

Most patients (63.4%) answered negatively to the question inquiring whether or not they had seen the charter of patient rights, and only 36.6% had seen the charter. As for the source of information on the contents of the bill, out of 36.6% who had responded positively, most (28.3%) mentioned the hospital as their source and only 1.5% had received any information from public media.

In the second part of the questionnaire, the results indicate that the awareness score ranging from 0 to 10 had a mean value of 3.226 and a standard deviation of 3.68. Most patients (58.9%) had poor knowledge, 12% had intermediate knowledge, and 29.1% had good knowledge (Table 1).

**Table 1:** Awareness of admitted patients of the bill of patient rights in 2011

Awareness	Count	Percent
Good (7-10)	98	29.1
Intermediate (4-6)	40	12
Poor (0-3)	198	58.9
Total	336	100

Regarding the content of the bill, the mostly ignored article was the right to participate in or abstain from a research activity (84.8%). Moreover, 60.4% were not aware of articles 1 (the right to receive treatment with dignity) and 4 (the right to receive information about the adverse effects of therapy). As table 2 depicts, the most unrecognized articles in decreasing order of unawareness included article 10 (the right to learn about insurance coverage and expenses if transferred to other centers) with 84.2% unawareness, article 8 (the right of access to physicians, etc during admission and after discharge) with 70.5% unawareness, article 7 (the right for privacy by the physician and others and giving permission) with 66.4% unawareness, article 6 (the right of confidentiality of personal information) with 65.2% unawareness, article 2 (the right to know the names of physicians, nurses etc) with 63.4% unawareness, article 5 (the right of discharge at any time with free will) with 61.3% unawareness, and article 3 (the right to learn about the process of therapy and the course of the disease) with 60.7% unawareness.

**Table 2:** Distribution of frequency of patient awareness of the bill of patient rights in 2011

Patient Rights	Aware	Unaware
1- right of anonymous treatment	133 (39.6)	203 (60.4)
2- right to know the name of physicians, nurses, etc	123 (36.6)	213 (63.4)
3- right to know about the treatment process and course of disease	132 (39.3)	204 (60.7)
4- right to know about complications of therapy and participation in treatment	133 (39.6)	203 (60.4)
5- right of discharge with free will	130 (38.7)	206 (61.3)
6- right of privacy and confidentiality of information	117 (34.8)	219 (65.2)
7- right of confidentiality by physicians and permission	113 (33.6)	223 (66.4)
8- right of access to physicians, etc during admission and after discharge	99 (29.5)	237 (70.5)
9- right to participate in or refrain from a research	51 (15.2)	285 (84.6)
10- right to learn about insurance coverage and expenses when transferred to other healthcare centers	53 (15.8)	283 (84.2)

Numbers in parentheses are expressed as percent.

In addition, we found a significant relationship between awareness scores and the years of education using Pearson’s correlation coefficient (r=0.3, p<0.0005). r^2^=0.09 indicates that some 10% of changes in the mean awareness scores is related to the patients’ years of education. Furthermore, Pearson’s correlation coefficient revealed a significant relationship between awareness cores and the answer to the questions, “Have you seen the charter of patient rights?” (r=0.809, p<0.0005). In other words, about 65% of changes in the awareness scores of patients are related to whether or not the patients have seen the charter in the hospital.

Another finding of our study is the inverse relationship between participants’ age and their awareness scores (r=0.32, p<0.0005). Independent t-test indicated that this relationship is significant, (t=49.89, p=0.0005) with 95% confidence. Moreover, we found no significant relationship between awareness score and variables of sex, length of stay, and type of insurance.

## 4. Discussion

Our findings indicate that most patients (63.4%) had not seen the bill of patient rights and only 36.6% mentioned having seen the bill. Kuzu (2006) reported that in Turkey, only 9% of patients were aware of the regulations of patient rights. In Russia, Fotaki (2006) reported that half of the respondents did not know that there are clear rights and credits for patients. Another study by Deneyer et al in Flemish indicated that only 7.8% of pediatricians were well aware of these regulations (Deneyer, 2012). In Turkey, Ozdemir et al. (2006) concluded that 40% of participating physicians were not aware of legal issues and 63% had never read anything about the legal issues surrounding patient rights. In Iran, Hamadan, 56.2% of patients mentioned that they were not familiar with the bill of patient rights and only 29.3% were aware of it (Hojjatoleslami, 2012). Another study by Hakan Ozdemir et al. (2011) indicated only 34% of participants (midwives and nurses) in university hospitals, state hospitals and village clinics, knew any legal basis for patients’ rights. In Lithuania Ducinskiene et al. (2006) indicated 85% medical staff 56% patients had heard or read about the law on patient rights. They suggest a need for awareness-raising among patients.

In Shanghais psychiatric hospitals, 52% medical staff had not education in ethics while almost all (89/1%) thought it was necessary. They reported (87.8%) that their medical institutions had not Ethics Committee. Su et al. (2008) reported (sample 1094), only 11% and 16.6 % respectively knew of the Nuremberg Code and the Declaration of Helsinki.

This factor requires further attention Physicians, nurses, and other members of the healthcare team should attend classes of ethics and law to gain the professional information required for new situations. As for patients, education may be accomplished on admission or any other suitable time, through provision of information both orally and in written (via pamphlets, brochures, booklets, etc). In the teaching hospitals in our study, the bill of patient rights was placed on the walls of all wards; however, many patients fail to read and comprehend it for many reasons. It has even been observed that some senior nursing and midwifery students are not properly aware of patient rights. The problem seems to lie in communications, and hospital staff needs to spend more time communicating with patients. The authorities may also consider compensatory rewards for encouraging their personnel.

Another finding in the present study is that the majority of participants (28.5%) received their information from the hospital and only 1.5% was informed via public media about the bill of patient rights. As patients and their companions regard sufficient information as one factor contributing to the observance of their rights and Patient satisfaction could prove a useful right to health indicator (Mpinga, 2011). The need for information provided by hospitals and other healthcare centers, as well as public media including the radio and television (which play an important role in Iranian common culture) is highlighted.

The findings of the present study indicate that 58.9% of participants had poor knowledge, 12% had intermediate knowledge, and 29.1% had good knowledge. Zeina et al. (2013) in South Egypt, reported the most patients (three quarter) and companions did not know about the list of patients’ rights.

Poor knowledge of the charter is not limited to patients, as Ghodsi and Hojjatoleslami (2012) reported students’ awareness in a hospital in Hamadan to be poor in 31%, intermediate in 53% and good in 16%. Considering these reports on the awareness of patients, managers and students reveals that despite the fact that the charter of patient rights has been developed in 2002 and revised on 2009, members of the treatment teams in healthcare centers are still not properly educated about patient rights. Resolving this shortcoming requires joint efforts by authority at all managerial levels. It must be noted that some hospitals affiliated with Mazandaran University of Medical Sciences are conducting projects to establish clinical governance, and we hope to observe the results in surveys of coming years.

Another finding of ours is related to the awareness of the contents of the charter. The least awareness pertained to the right of participating in or abstaining from research activities, mentioned in article 9 of the charter (15.2%), while the best awareness pertained to the two domains of treatment with dignity (article 1) and knowledge of complications of therapy (article 4) with 39.6% awareness.

The right to equal treatment, irrespective of age, gender, ethnicity, socio-economic status and place of residence, is an important for several health care system (Askildsen, 2010). In modern medical ethics, great emphasis is placed on the principle of respect for patient autonomy. Patients are now the ultimate decision –makers (Rowe, 2013). In Austria, Stadlbauar et al, an online survey among 3 groups (ICU nurses n=185;students of health sciences n=1277; students of non- health science related courses n=485) showed that they know (84%) the Austrian organ donation legislation (Stadlbauer, 2013).

On the other hand, Krzych and Ratajczyk (2013) in Poland reported that over 80% of respondents were aware of their right to choose a treating physician, refusal of the proposed treatment, the choice of the place where the patient is treated, the right of access to medical records, free meals, pastoral care, ability to provide to third parties information about the state of health, as well as giving information to particular persons by phone. The least awareness was shown in relation to the form of giving consent (42.9%) or refusal of consent (50.5%) to treatment and the doctors’ right to refuse the presence of a person close to the patient during treatment (16.4%).

The above results indicate that patients do not have appropriate knowledge of article 9 of the charter of patient rights (dealing with the right to participate in or abstain from a research). In Lithuania, information about side-effects to patients is not accordance with the principle of the respect for patients’ autonomy and requirements of Lithuanian (Liseckiene, 2008)

Considering the poor knowledge of article 9, those in charge at hospitals and healthcare centers must familiarize patients with this article. In addition, other healthcare personnel must improve their knowledge through training programs for research ethics. It must be noted, however, that mere knowledge will not be sufficient. Mohammad Nejad et al. (2008) reported the knowledge rate of nurses employed in teaching hospitals of Tehran to be good (95.5%). It appears that knowledge alone cannot guarantee the observance of patient rights by the medical team. It is necessary that legislators should clearly define the punishments and other legal issues associated with disregard of each article in the charter to grant a legal and executive guarantee to the charter.

In Turkey, Kuzu reported (2006) that the most common causes of not requesting appropriate service are fearing the anger of healthcare personnel (55.7%), concerns over the negative impact on treatment process (20%), poverty, illiteracy, physical problems, immigration, and timidity (14%), unawareness of the regulations (5.8%), and overworking of personnel and poor communication between patients and personnel (4.3%). It seems that in addition to knowledge of patients and personnel and legal measures, other factors (such as social culture) affect the observance of patient rights.

Regarding other articles of the charter, 60.4% were not aware of the 4^th^ article, dealing with the right to choose the final therapy. In Turkey, Erer (2008) reported that only 43.3% of cancer patients knew that they could reject the therapy proposed by the physician. Arab et al. (2010) reported that the knowledge of hospital managers in the public and private hospitals regarding patient autonomy was 23% and 57%, respectively which falls in the poor category. This indicates that even hospital managers, who are expected to be better aware of the charter than others, have poor knowledge of the matter.

Awareness of the charter of patient rights is vital for patients and finding of our study is the significant relationship between mean score of awareness and years of education, which is consistent with the findings of 10, 27.

Unfortunately, most patients in our study were illiterate or below high school (61%). As Table 3 depicts, most patients with poor knowledge scores were illiterate or educated below high school (approximately 41.9%). This indicates that education influences the patients’ awareness.

**Table 3:** Awareness of contents of bill of patient rights and education

Education	Awareness			Total
	Poor	Intermediate	Good	
Illiterate	67 (19.9)	7 (2.1)	10 (3)	84 (25)
Below High School	74 (22)	16 (4.8)	31 (9.2)	121 (36)
High School	37 (11)	13 (3.9)	38 (11.3)	88 (26.2)
College	9 (2.7)	3 (9)	2 (6)	14 (4.2)
Bachelor	198 (58.9)	40 (11.9)	98 (29.2)	336 (100)

Another finding in the present study (Table 4) is the fact that individuals who had not seen the charter tended to have poor scores (about 56%), whereas those who had seen the charter tended to have good scores (26.2%). This finding indicates that the managers must improve the visibility of the charter of patient rights. Currently, the charters are placed on walls of the wards, which seem to be less than satisfactory. We recommend that more efficient methods, such as pamphlets, brochures, and booklets, should be submitted to patients alongside oral explanations on admission.

**Table 4:** Awareness of contents of bill of patient rights and seeing the charter

Seeing	Awareness			Total
	Poor	Intermediate	Good	
Seen	188 (56)	15 (4.5)	10 (3)	213 (63.4)
Not Seen	10 (3)	25 (7.4)	88 (26.2)	123 (36.6)
Total	198 (58.9)	40 (11.9)	98 (29.2)	336 (100)

Yaghobian et al. (2009) conducted a quasi-experimental study to conclude that using educational texts alone will not be sufficient for nurses with bachelor’s degree, and a combination of texts and lectures will be more efficient. Evidently, illiterate patients and those with lower levels of education require further help for comprehension of the subject, and the material should be presented in a simple and comprehensible fashion.

The source of information about the charter is another finding of our study. As presented in Table 5, most of those who had seen the charter (28.3%) had done so in the hospital, whereas only 1.5% of them had learned about it via public media. Arab et al. (2010) reported that only 13.2% of patients had heard about patient rights, and 8.5% had read about the matter. These findings indicate that authorities need to pay extra attention to public media and the role they may play in improving the awareness of the public.

**Table 5:** Awareness of contents of bill of patient rights and information source

Source	Awareness			Total
	Poor	Intermediate	Good	
Friends and Acquaintances	1 (3)	6 (1.8)	12 (3.6)	19 (5.7)
Public Media	2 (6)	1 (3)	2 (6)	5 (1.5)
Hospital	6 (1.8)	19 (5.7)	70 (20.8)	95 (28.3)
Not seen	189 (56.3)	14 (4.2)	14 (4.2)	217 (64.6)
Total	198 (58.9)	40 (11.9)	98 (29.2)	336 (100)

Another finding is the inverse relationship between awareness scores and the patients’ age. Arab et al. (2010) and Krzych and Ratajczyk (2013) reported significant relationships between age and awareness scores. Considering the mean age of patients in our study (41 years) which is similar to that reported by Arab et al. (2010) (43.6 years) it is crucial to pay attention to the education of middle-aged and senile patients. Moreover, we did not observe a significant relationship between awareness scores and the variables of sex, type of insurance and length of stay. Similarly, Moghaddam et al. (2011) failed to find a significant relationship between length of stay and awareness of the charter whereas Arab et al. (2010) reported a significant relationship between these two variables.

It must be noted that the mean length of stay was 4.6 ± 3.24 days in our study and 7.1 ± 7.4 days in that of Arab et al. It is possible that the longer stay in the latter may have influenced the patients’ awareness.

## 5. Conclusion

The findings of the present study indicate that about 70% of patients did not have good knowledge of the contents of the bill of patient rights, and only 36.6% had seen the charter. Therefore, we have the following recommendations:

1- Placing the bill in public areas of healthcare centers, especially in places where the patients and their companions await admission or other services
2- Preparing brochures, pamphlets, or booklets in a simple language and submitting them to the patient on admission
3- Preparing regulations with executive guarantees
4- Establishing ethics committees in healthcare centers with attorneys and professionals well aware of patient rights
5- As nurses, particularly head nurses, are in direct contact with patients and are among the first people to notice the observance of patient rights, educating them about the regulations will render them defenders of patient rights in wards. The awareness of head nurses is necessary, but not enough. Ethics committees in hospitals may grant a certain degree of authority to hospital nurses and supervisors.
6- Just in the same way that mothers are routinely educated about breastfeeding in healthcare centers, so must all patients be routinely informed about the contents of the bill of patient rights.
7- Healthcare centers often face the challenge of shortages in educating personnel. Patient education may be accomplished with help of students at all levels of education and with different fields. This is not only cost effective and less time consuming, but also serves as a reminder to students to learn about and propagate the bill of patient rights.

## Study Limitations

1- The current study was conducted in teaching hospitals only, and gives no knowledge of the situation in private hospitals.
2- We did not recruit any patient from the emergency department or oncology, pediatrics and psychiatric wards. Thus, we propose the future studies to cover these points, as well.

## Ethical Consideration

The study was approved by the research center for Traditional and Alternative Medicine and Study Center for Medical Ethics and History Mazandaran University of Medical Sciences.

The researchers contacted the administrator in each of the 4 hospitals in order for data collection to be carried out; the study was explained and permission to enter the hospital for the purpose of gathering data was sought.

## References

[ref1] Arab M, Zarei A, Hosseini M (2010). Awareness and Observation of Patients Rights from the Perspective Of Patients: A Study In University Hospitals In Tehran. Journal of Public Health, School of Public Health.

[ref2] Askildsen J. E, Holmås T. H, Kaarboe O (2010). Prioritization and patients’ rights: Analysing the effect of a reform in the Norwegian hospital sector. Social Science & Medicine.

[ref3] Bassiri Moghadam K, Bassiri Moghadam M, Moslem A, Ajam Zybd H, Gamal F (2011). Awareness of Patients And Medical Staff And Patient’s Rights In Respect Of Teaching Hospitals Gonabad. Journal of Knowledge of ogh.

[ref4] Deneyer M, Marchand J, Buy R, Michel L, Holsters D, Vandenplas Y (2012). The influence of the law on patient’s rights on the practice of the Flemish paediatricians anno 2010. Acta chirurgica Belgica.

[ref5] Ducinskiene D, Vladickiene J, Kalediene R, Haapala I (2006). Awareness and practice of patient’s rights law in Lithuania. BMC Int Health Hum Rights.

[ref6] Erer S, Atici E, Erdemir A (2008). The views of cancer patients on patient rights in the context of Information and Autonomy. Journal of Medical Ethics.

[ref7] Fehime Zülfikar M, Ulusoy F (2001). Are Patients Aware of Their Rights?. A Turkish study Nurs Ethics.

[ref8] Fotaki M (2006). Users’ perceptions of health care reforms: quality of care and patient rights in four regions in the Russian Federation. Social Science & Medicine.

[ref9] Ghodsi Z, Hojjatoleslami S (2012). Knowledge of students about Patient Rights and its relationship with some factors in Iran. Procedia - Social and Behavioral Sciences.

[ref10] Hojjatoleslami S, Ghodsi Z (2012). Respect the rights of patient in terms of hospitalized clients: a cross- sectional survey in Iran, 2010. Procedia - Social and Behavioral Sciences.

[ref11] Humayun A, Fatima N, Naqqash S, Hussain S, Rasheed A, Imtiaz H, Imam S. Z (2008). Patients’ perception and actual practice of informed consent, privacy and confidentiality in general medical outpatient departments of two tertiary care hospitals of Lahore. BMC Med Ethics.

[ref12] Joolaee S, Nasrabadi A. N, Yekta Z. P, Tschudin V, Mansouri I (2006). An Iranian Perspective on Patients’ Rights. Nurs Ethics.

[ref13] Joolaee S, Tschudin V, Nikbakht-Nasrabadi A, Parsa-Yekta Z (2008). Factors affecting patients’ rights practice: the lived experiences of Iranian nurses and physicians. Int Nurs Rev.

[ref14] Kazemnezhad Mahmood S, Hesamzadeh A (2013). Implementation of patients’ bills of rights by physicians and nurses from their colleagues’ points of view in educational hospitals of Mazandaran University of medical sciences. Journal of Mazandaran University of Medical Sciences.

[ref15] Krzych Ł. J, Ratajczyk D (2013). Awareness of the patients’ rights by subjects on admission to a tertiary university hospital in Poland. Journal of Forensic and Legal Medicine.

[ref16] Kuzu N, Ergin A, Zencir M (2006). Patients’ awareness of their rights in a developing country. Public Health.

[ref17] Liang Sua B, Huanga J, Mellorc D, Yanga W, McCabec M, Shena Y, Xu Y (2012). The rights of psychiatric patients in China: A survey of medical staff and consumers’ attitudes toward patient participation in clinical trials. Social Science & Medicine.

[ref18] Liseckiene I, Liubarskiene Z, Jacobsen R, Valius L, Norup M (2008). Do family practitioners in Lithuania inform their patients about adverse effects of common medications?. J Med Ethics.

[ref19] Millar M (2011). Patient rights and healthcare-associated infection. J Hosp Infect.

[ref20] Mohammad Nejad E, Begjani J, Abotalebi G, Salari A, Ehsani S. R (2011). Nurses awareness of patients rights in a teaching hospital. J Med Ethics Hist Med.

[ref21] Mossadeghzadeh Mohammad A (2006). The Relationship Between Patients ‘Knowledge Of Patients’ Rights And Their Satisfaction With Hospital Services. Teb And Tazkiyeh.

[ref22] Mpinga E. K, Chastonay P (2010). Satisfaction of patients: a right to health indicator?. Health Policy.

[ref23] Ozdemir M. H, Ergönen A. T, Sönmez E, Can I. O, Salacin S (2006). The approach taken by the physicians working at educational hospitals in Izmir towards patient rights. Patient Education and Counseling.

[ref24] Parsapour A, Bagheri A, Larijani M. A (2010). Bill of Patients’ Rights in Iran. Iranian Journal of Medical Ethics and History of Medicine.

[ref25] Rowe K, Moodley K (2013). Patients as consumers of health care in South Africa: the ethical and legal implications. BMC Med Ethics.

[ref26] Stadlbauer V, Steiner P, Schweiger M, Sereinigg M, Tscheliessnigg K. H, Freidl W, Stiegler P (2013). Knowledge and attitude of ICU nurses, students and patients towards the Austrian organ donation law. BMC Med Ethics.

[ref27] Su L, Huang J, Yang W, Li H, Shen Y, Xu Y (2012). Ethics, patient rights and staff attitudes in Shanghai’s psychiatric hospitals. BMC Med Ethics.

[ref28] Välimäki M, Kuosmanen L, Kärkkäinend J, Kjervike D. K (2009). Patients’ rights to complain in Finnish psychiatric care: An overview. International Journal of Law and Psychiatry.

[ref29] Yaghobian M, Yaghobi T, Salmeh F, Golmohammadi F, Safari H, Savasry R, Habibi Kh (2009). Comparison with Booklet and Lecture Teaching Methods on Nurses’ Knowledge About The Laws And Professional Regulations. Iranian Journal of Medical Education/Winter.

[ref30] Zeina H. A, El Nouman A. A, Zayed M. A, Hifnawy T, El Shabrawy E. M, El Tahlawy E (2013). Patients’ rights. J Empir Res Hum Res Ethics.

